# Descriptive Analysis of Perioperative Mortality Rates Following Major Amputations: A 15-Year Retrospective Study at a Teaching Hospital

**DOI:** 10.7759/cureus.113312

**Published:** 2026-07-24

**Authors:** Henrique Jose Pereira de Godoy, Jose Maria Pereira de Godoy, Thaisa Bonardi, Milton Sergio Bohatch Junior, Selma Regina de Oliveira Raymundo

**Affiliations:** 1 Vascular Surgery, Faculty of Medicine of São José do Rio Preto (FAMERP), São José do Rio Preto, BRA; 2 Vascular Surgery, Clínica Godoy-Sao Jose do Rio Preto, São José do Rio Preto, BRA; 3 Vascular Surgery, Faculty of Medicine of São José do Rio Preto (FAMERP), Sao Jose do Rio Preto, BRA; 4 Angiology and Vascular Surgery, Clínica Godoy-Sao Jose do Rio Preto, Sao Jose do Rio Preto, BRA

**Keywords:** diabetes, major amputations, mortality, peripheral vascular disease, population study, survival analysis

## Abstract

Background

Lower limb amputations are frequently preceded by diabetic foot ulcerations. When these wounds are complicated by progressive bacterial infections, they can rapidly advance to moderate or severe stages, significantly elevating the necessity for limb amputation, reflecting in mortality risk. This study aimed to evaluate the perioperative mortality rate of major lower limb amputations between 2010 and 2024 at a teaching hospital.

Methodology

A retrospective study was conducted by reviewing the medical records of patients who underwent non-traumatic major amputations (transtibial and transfemoral) at the Hospital de Base (HB) in São José do Rio Preto between 2010 and 2024. The hospital is the second-largest teaching hospital in Brazil, serving as the clinical and teaching hospital for the Faculty of Medicine of São José do Rio Preto (FAMERP). It is a premier tertiary facility, heavily recognized for its intensive care medicine, cardiology, and trauma surgery. The study analyzed perioperative mortality rates stratified by age and sex. Statistical analysis was performed using descriptive methods to evaluate trends and demographic distributions over the 15-year period.

Results

A total of 1,744 patients were identified. The average age per year ranged from 61.56 to 68.8 years (mean = 64.82 years). Mortality occurred in 123 (19.46%) of 632 female patients and in 204 (18.21%) of 1,112 male patients (chi-square test: p-value = 0.56). Regarding deaths, there were 327 in total, varying by year from 11.45% to 25.5%, with 2021 being the most critical year, the main year of COVID-19 at the institution. The mean mortality rate was 19.02%. Before COVID-19 in 2010-2019, there were 234 deaths in 1,108 patients (20.1%), and during the COVID-19 period (2021 and 2022), there were 75 deaths in 408 patients (18.4%), with no statistically significant difference.

Conclusions

Mortality rates for non-traumatic patients undergoing major amputations from the emergency room admission remained high, making it necessary to address the issue as early as possible to prevent sepsis, the leading cause of mortality, and to improve prevention, especially for patients with diabetes, which was the most significant cause associated with amputation.

## Introduction

Around 50-60% of ulcers become infected, with approximately 20% of moderate-to-severe infections resulting in lower limb amputation [1]. Consequently, roughly 80% of lower limb amputations are associated with the complications of diabetes mellitus and foot ulceration, both of which significantly elevate the risk for morbidity and mortality. The six-year mortality rate following a major amputation is 72%, underscoring the clinical severity of this condition [2]. Furthermore, evidence indicates that hospitalized patients with diabetic foot complications experience a 12.5% rate of adverse cardiovascular events and a 4.4% mortality rate. Notably, cases involving distal arterial obstructions require more frequent vascular interventions than those involving other pathological patterns [3].

The one-year mortality rate for diabetic patients undergoing hemodialysis is 33.8%, compared to 17.6% for those not on dialysis. At three, five, and seven years, mortality rates remain significantly higher in the hemodialysis group (58.8%, 69.9%, and 87.6%, respectively) than in the non-dialysis group (37.0%, 50.5%, and 64.4%, respectively). Furthermore, the median survival time is significantly shorter for patients on hemodialysis at 27.1 months, compared to 47.7 months for those not on dialysis (p < 0.001) [4]. Independent predictors of major amputation include the presence of extensive pedal arterial occlusive disease (desert foot), persistent post-procedure pain, heel involvement with multiple ulcers, and the inability to stand or walk without assistance [5].

One study suggests that while endovascular and open limb revascularization procedures do not significantly impact major amputation or mortality rates, bypass surgery is associated with a significantly lower postoperative reintervention rate [6]. Currently, there is no evidence to reliably conclude that angioplasty with conventional stenting, drug-eluting balloon angioplasty, or atherectomy is superior to conventional balloon angioplasty in preventing major amputations and deaths in patients with chronic limb-threatening ischemia [7].

Regarding lifestyle and demographic factors, former smokers undergoing lower limb revascularization show five-year outcomes similar to non-smokers, with better overall and progression-free survival compared to active smokers [8]. Gender-based differences are also observed. Women are more likely to die within 30 days, whereas men have higher rates of reintervention; however, limb salvage rates are higher in women [9]. Additionally, frailty independently predicts short- and long-term all-cause mortality, though not with major amputations, in patients with peripheral arterial disease, serving as a critical factor for risk stratification [10].

This study aimed to evaluate perioperative mortality following major lower limb amputations performed between 2010 and 2024 at the Hospital de Base of the Faculty of Medicine of São José do Rio Preto (FAMERP), Brazil.

## Materials and methods

Study design and setting

This is a retrospective analysis of a prospectively maintained database, evaluating perioperative mortality rates for major lower limb amputations. The research was conducted at the Hospital de Base of the FAMERP, São Paulo, Brazil, covering the period between January 2010 and December 2024.

Inclusion and exclusion criteria

Medical records of patients undergoing above-knee or below-knee non-traumatic major lower limb amputations by the vascular surgery service between January 2010 and December 2024 were considered for inclusion. Medical records of patients who underwent minor amputations (e.g., toes, transmetatarsal) and cases where the amputation indication was primarily due to orthopedic, neoplastic, or traumatic conditions were excluded.

Data collection

Data was extracted from the hospital’s electronic database and organized into a dedicated Microsoft Excel spreadsheet (Microsoft Corp., Redmond, WA, USA). The primary variables analyzed included perioperative mortality, biological sex, and age.

Statistical analysis

A descriptive statistical analysis was performed to evaluate mortality rates by year, sex, and age group (Yates-corrected chi-square: p-value = 0.5), comparing two groups: the COVID-19 and non-COVID-19 period. Statistical analyses were performed using StatsDirect software (Version 4.0.4; StatsDirect Ltd., Cheshire, UK).

Ethical considerations

The study protocol was reviewed and approved by the Research Ethics Committee of the FAMERP, Brazil (approval number: 7.346.790). Patient consent for publication was exempted by the Research Ethics Committee due to the retrospective design, the historical nature of the medical records, and the impossibility of contacting family members of deceased patients. Thus, the authors ensured the privacy and confidentiality of the data obtained, fully preserving the anonymity of the participants in accordance with the Declaration of Helsinki.

## Results

**Figure 1:**
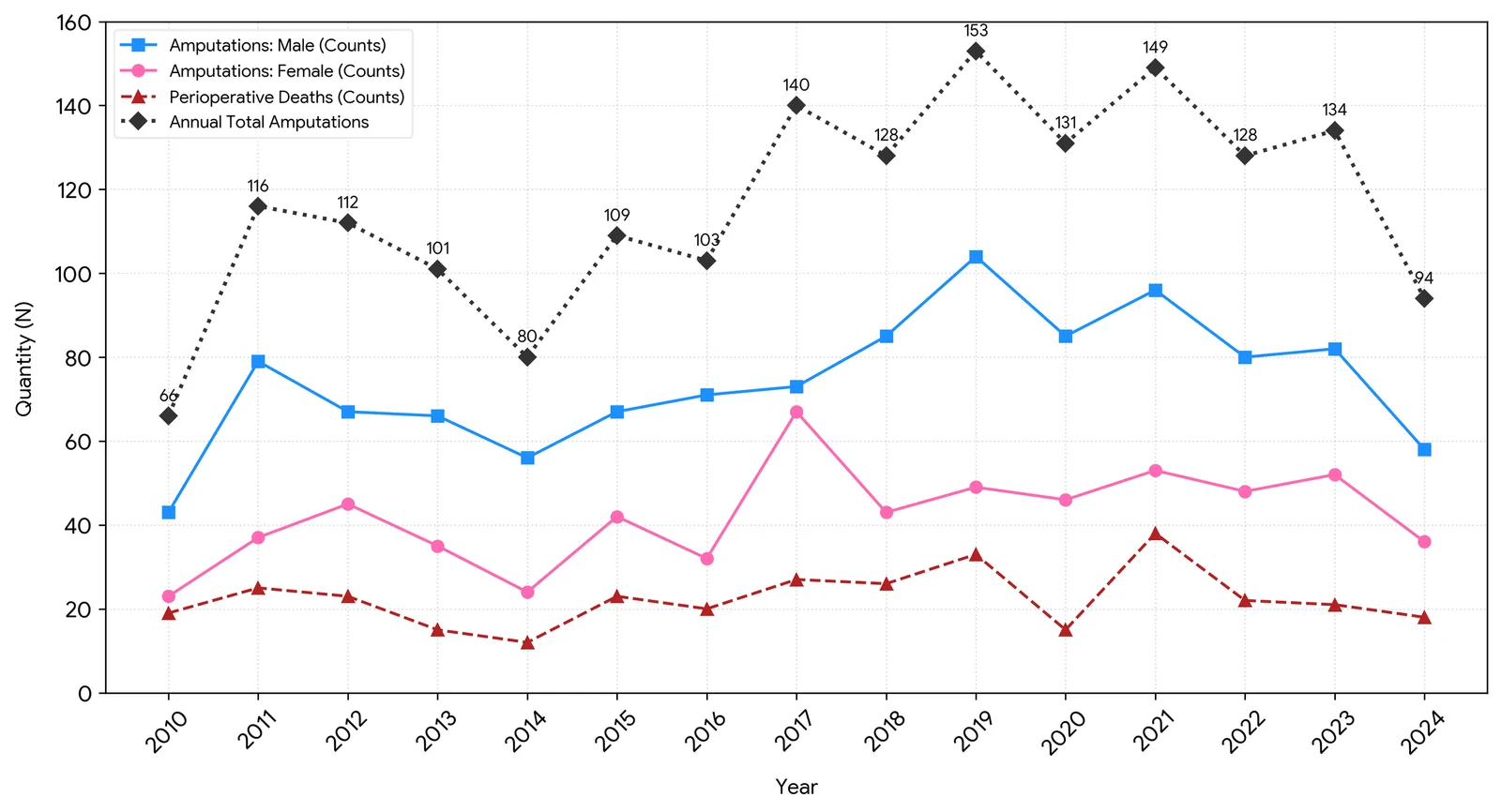
Time-series analysis of amputation counts and perioperative mortality between 2010 and 2024. The graph illustrates the temporal variation in the absolute quantity (N) of amputations among male patients (blue line with squares) and female patients (pink line with circles), compared to the number of perioperative deaths (red dashed line with triangles). Additionally, the combined annual total of amputations is presented (black dotted line with diamonds) with explicit numerical values displayed for each year.

**Figure 2:**
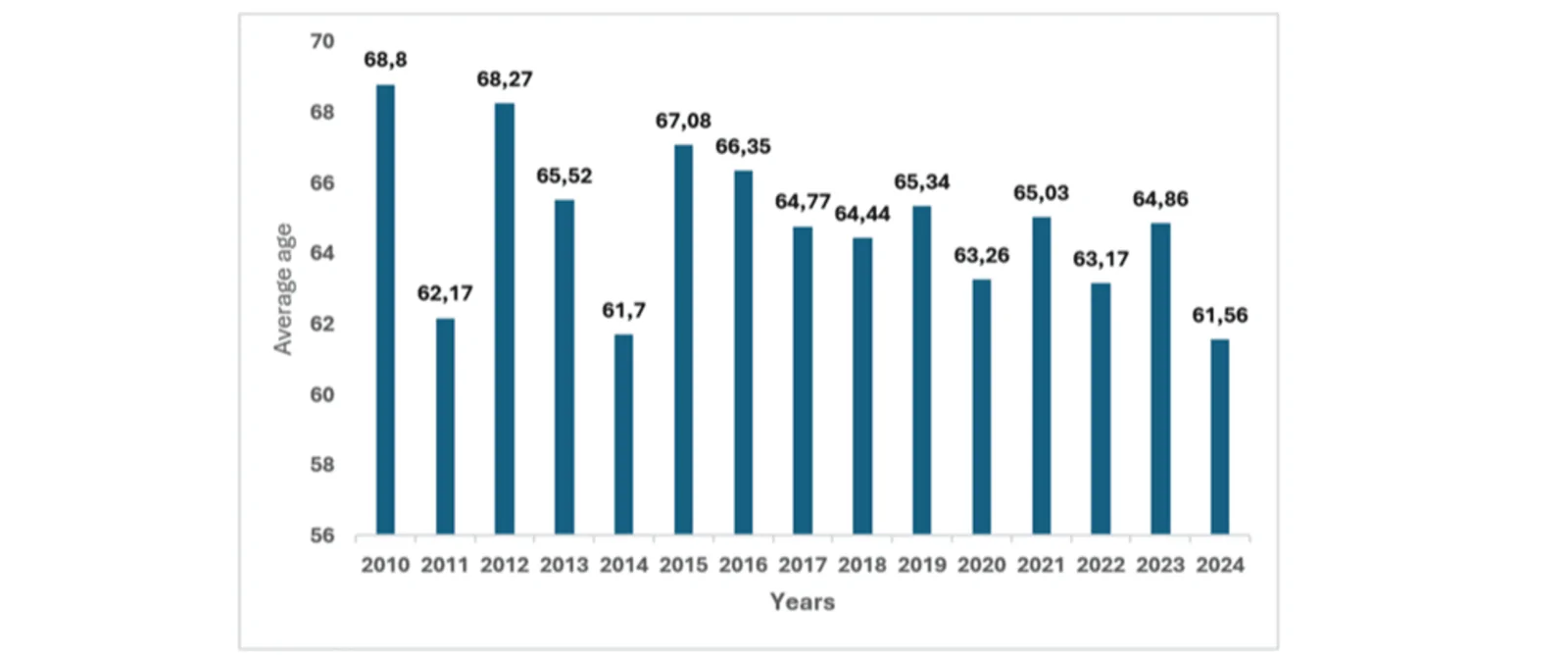
Annual trends in the mean age of patients undergoing major amputation.

**Figure 3:**
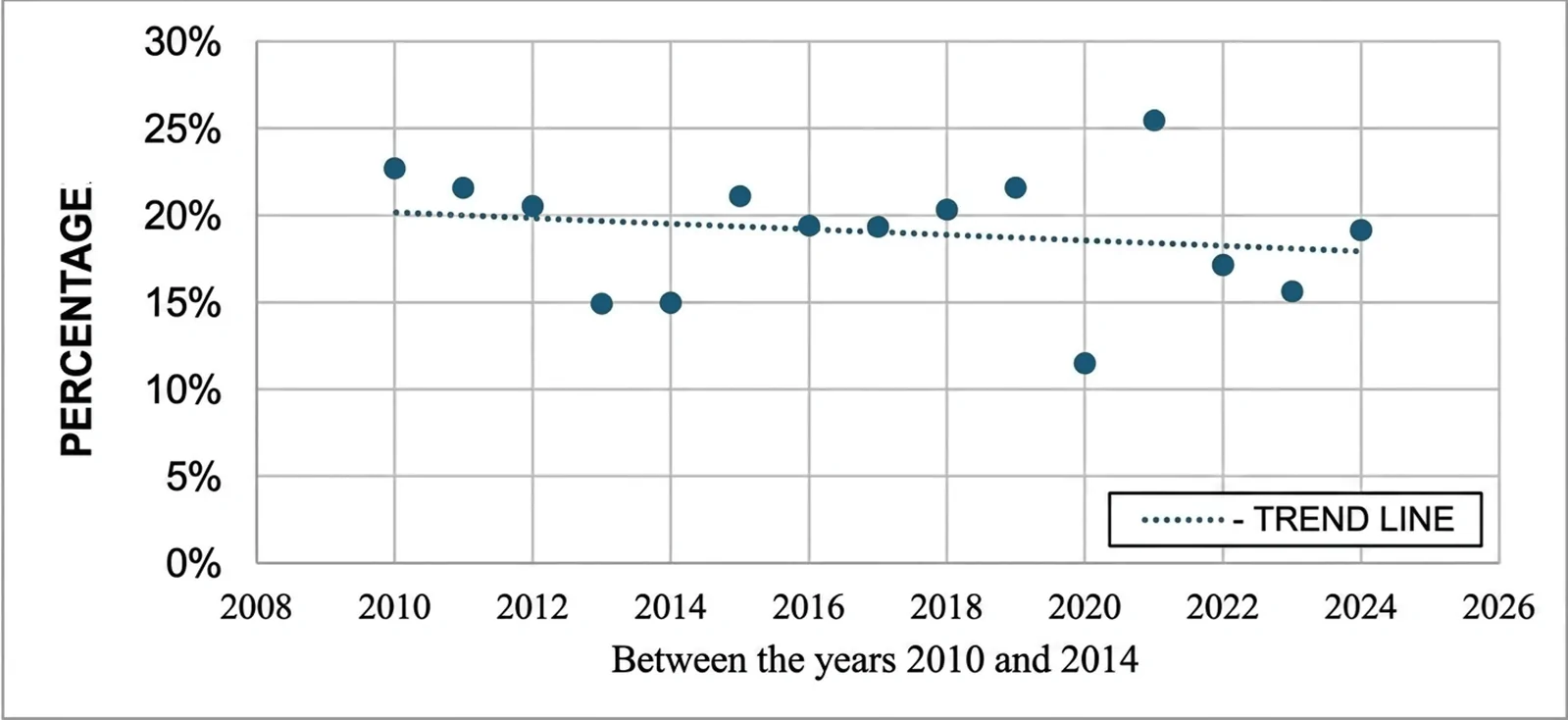
Percentage of deaths per year of patients undergoing major amputation.

A total of 1,744 patients were identified, as summarized (descriptive analyses) in Table 1 and Figure 1. The annual average age ranged from 61.56 to 68.8 years, with an overall mean of 64.82 years (SD = 16.45; median = 67 years) (Figure 2). Mortality occurred in 123 (19.46%) of 632 female patients and 204 (18.34%) of 1,112 male patients; this difference was not statistically significant (chi-square test: p = 0.56). A total of 327 deaths were recorded, with annual mortality rates ranging from 11.45% to 25.5%. The highest mortality rate occurred in 2021, which was the period most affected by the COVID-19 pandemic at the institution. The mean annual mortality rate was 19.02%. During the non-COVID-19 period (2010-2019), 234 deaths occurred among 1,108 patients (21.12%), while during the pandemic period (2021-2022), there were 75 deaths among 408 patients (18.38%) (Figure 3). No statistically significant difference (Yates-corrected chi-square test: p = 0.5) was observed between these two groups (COVID-19 and non-COVID-19 groups).

**Table 1:** Annual distribution of major amputations stratified by sex and perioperative mortality rates.

Year	Amputations, female	Amputations, male	Amputations, Total	Perioperative deaths (N)	Perioperative deaths (%)
2010	23	43	66	19	28.79
2011	37	79	116	25	21.55
2012	45	67	112	23	20.54
2013	35	66	101	15	14.85
2014	24	56	80	12	15.00
2015	42	67	109	23	21.10
2016	32	71	103	20	19.42
2017	67	73	140	27	19.29
2018	43	85	128	26	20.31
2019	49	104	153	33	21.57
2020	46	85	131	15	11.45
2021	53	96	149	38	25.50
2022	48	80	128	22	17.19
2023	52	82	134	21	15.67
2024	36	58	94	18	19.15
Total	632	1,112	1,744	337	19.32

## Discussion

A previous study at this institution evaluating mortality after major lower limb amputation for critical ischemia between 2005 and 2007 reported a 15.6% mortality rate within one month, with 5.6% occurring within the first week [11]. The findings of the present study indicate that there has been no significant change in perioperative mortality over these two decades of evaluation. For instance, mortality rates were reported at 44% in 2005 and 47.5% in 2019, demonstrating a lack of reduction between these two periods [12].

This persistently high mortality rate highlights the clinical severity of the condition and the complexity of its management. A secondary consideration is the deterioration in the quality of life for both patients and their caregivers, who frequently require specialized postoperative support [13,14]. Given these high mortality rates, quality of life must be integrated as a primary criterion in the decision-making process regarding limb revascularization versus amputation.

The primary causes of mortality in individuals in their sixth decade are typically cardiac and neurological events associated with atherosclerosis. However, these were not the leading causes of perioperative mortality in the present study. Instead, a significant number of deaths were related to factors that could potentially be mitigated through improved infection control and earlier surgical intervention before progression to systemic sepsis [15]. Furthermore, rehospitalization due to infection of the amputation stump was a frequent outcome observed during the study period, occurring in some cases despite the administration of prophylactic antibiotic therapy [16].

Over the years, several studies have been conducted to identify the primary factors leading to infection and sepsis, which subsequently increase mortality. Hospital de Base is a tertiary facility that receives patients from numerous smaller municipalities, serving a regional population of approximately 1.7 million people. However, vascular surgeons are not always available in these smaller towns to assess peripheral circulation; consequently, management is often limited to antibiotic therapy. Our findings indicate that approximately 33% of these cases involve severe ischemia requiring revascularization. Patients treated solely with antibiotics frequently experience clinical deterioration and are referred to the tertiary center at an advanced stage of their disease. Furthermore, between 85% and 90% of these patients with ulcers are diabetic, and infections involving Gram-negative bacteria are common, resulting in a mortality rate exceeding 20% [17,18].

Our data revealed that approximately 60% of patients with initially negative radiographic findings had positive cultures. This discrepancy led to the implementation of repeat foot radiography approximately 15 days post-amputation to reassess for osteomyelitis [18]. It is important to note that while plain radiography is not the gold standard for detecting pedal osteomyelitis, its universal availability in all municipalities makes it a viable tool. Adopting a strategy of repeat imaging 15 days after the initial assessment can achieve a diagnostic accuracy exceeding 90%.

Regarding the assessment of arterial circulation, performing an arterial and venous color Doppler ultrasound of the affected limb is the standard institutional protocol and is always considered for patients with peripheral artery disease. However, because this 15-year registry focuses on major amputations performed in an emergency setting, a massive proportion of these patients presented with acute, rapidly progressive infections, gas gangrene, or advanced sepsis. In these critical, life-threatening scenarios, immediate surgical intervention via emergency amputation to control the infectious focus was the absolute clinical priority to prevent mortality, rendering preoperative vascular mapping with color Doppler secondary or logistically unfeasible for the entire cohort.

Another concerning finding is the trend toward a reduction in the mean age at death among these patients. Paradoxically, we are also increasingly identifying octogenarian and nonagenarian patients with chronic arterial disease who require revascularization or amputation [19]. Palliative care is recommended not only during the active phase of dying but throughout the disease trajectory, particularly as one-year mortality rates can reach approximately 50%. This figure is comparable to, or even exceeds, the mortality rates associated with many forms of cancer [17].

One study identified significant differences in mortality rates between diabetic foot treatment centers, likely attributable to the specialized care provided for diabetic foot ulcers. Such variations in care may be a critical factor in the differing rates of major lower limb amputations [18]. A similar trend was observed at our institution, suggesting an urgent need for improved prevention and earlier intervention in regional towns, particularly regarding chronic arterial disease associated with diabetes. Recent evidence indicates that patients who undergo revascularization attempts before major lower limb amputation have a lower probability of mortality at one and three years postoperatively, underscoring the importance of early intervention [19]. In the present study, however, the majority of patients presented at advanced stages when they were no longer candidates for limb-salvage revascularization, except in cases where the procedure was performed to optimize perfusion for a specific amputation level. Another aspect observed was the fragility of these patients, which can contribute to amputations and mortality.

Cox regression analysis demonstrated that the presence of Gram-negative bacteria; male sex; the mean Wound, Ischemia, and Foot Infection score; diabetes mellitus; and end-stage renal disease were independent risk factors positively associated with amputation [20]. Therefore, infection represents a critical complication that significantly impacts mortality rates and increases the likelihood of subsequent amputations.

The overall mortality rates following major amputation were reported as 47.9%, 61.3%, 70.6%, and 62.2% at one, two, three, and five years of follow-up, respectively [21]. Another study identified a 30-day mortality rate of 22% and a one-year rate of 44%. Notably, patients with renal disease faced a 77% mortality rate at five years, representing a 3.5-fold higher risk of death [22]. Early postoperative mortality rates vary between 4% and 22% for minor and major amputations; however, data regarding the influence of patient-related factors, such as age and comorbidities, remain limited [23]. A comprehensive literature review found that 30-day mortality rates following lower limb amputation ranged from 7.1% to 51.4%, with a mean rate of 16.45% across the analyzed studies [24]. Furthermore, it has been reported that prior revascularization of the amputated limb was absent in 26% of cases, with 30-day and one-year mortality rates reaching 14% and 34%, respectively [25].

Another important aspect is wound management and dressing protocols, which can influence major amputation rates but have not been shown to impact mortality. Hyperbaric oxygen therapy was administered to approximately 300 patients. While this intervention reduces the rate of major amputation, it does not significantly affect mortality [26]. Consequently, the development of regional guidelines and specialized training regarding appropriate dressing techniques is fundamental [27,28].

Study limitations

This study had a few limitations. There was a lack of detailed stratification and the inability to perform statistical adjustments or sensitivity analyses for certain crucial clinical variables. Notably, the exact staging of chronic kidney disease, which is a major public health problem affecting approximately 11.5% of adults and is strongly associated with mortality [29], as well as specific microbial profiles and infection severity, were not uniformly or systematically recorded across the 1,744 patients in this 15-year historical registry. Furthermore, recent literature emphasizes that post-amputation mortality is particularly elevated in older females and patients with chronic kidney disease, reinforcing the urgent need for comprehensive risk assessment and optimized perioperative management [30]. Consequently, a formal multivariable adjustment or quantitative staging analysis for chronic kidney disease was mathematically unfeasible in the current study and would introduce severe statistical bias. However, these specific clinical, demographic, and metabolic factors are currently being evaluated individually and prospectively in ongoing research by our group.

## Conclusions

Mortality rates among patients undergoing non-traumatic major amputations have remained persistently high over the last 15 years. This sustained burden is particularly pronounced among high-risk subgroups, including older females and patients with chronic kidney disease. These findings underscore the urgent need for comprehensive risk assessments and optimized perioperative management to improve survival outcomes. Ultimately, refining regional prevention strategies for diabetic foot complications and ensuring timely clinical intervention for ischemia are critical pathways to reducing these mortality rates.
